# A constructive approach to the epistemological problem of emergence in complex systems

**DOI:** 10.1371/journal.pone.0206489

**Published:** 2018-10-30

**Authors:** Alberto Pascual-García

**Affiliations:** 1 Centro de Biología Molecular “Severo Ochoa” CSIC-Universidad Autónoma de Madrid, Madrid, Spain; 2 Department of Life Sciences Imperial College London, Silwood Park, Ascot, United Kingdom; University of British Columbia, CANADA

## Abstract

Emergent patterns in complex systems are related with many intriguing phenomena in modern science. One question that has sparked vigorous debates is if difficulties in the modelization of emergent behaviours are a consequence of ontological or epistemological limitations. To elucidate this question, we propose a novel approximation through constructive logic. Under this framework, experimental measurements will be considered conceptual building blocks from which we aim to achieve a description of the microstates ensemble mapping the macroscopic emergent observation. This procedure allow us to have full control of any information loss, thus making the analysis of different systems fairly comparable. In particular, we aim to look for compact descriptions of the constraints underlying a dynamical system, as a necessary *a priori* step to develop explanatory (mechanistic) models. We apply our proposal to a synthetic system to show that the number and scope of the system’s constraints hinder our ability to build compact descriptions, being those systems under global constraints a limiting case in which such a description is unreachable. This result clearly links the epistemological limits of the framework selected with an ontological feature of the system, leading us to propose a definition of emergence strength which we make compatible with the scientific method through the active intervention of the observer on the system, following the spirit of Granger causality. We think that our approximation clarifies previous discrepancies found in the literature, reconciles distinct attempts to classify emergent processes, and paves the way to understand other challenging concepts such as downward causation.

## Introduction

“What urges you on and arouses your, you wisest of men, do you call it will to truth? Will to the conceivability of all being: that is what I call your will! You first want to make all being conceivable: for, with a healthy mistrust, you doubt whether it is in fact conceivable. But it must bend and accommodate itself to you! Thus will your will have it. It must become smooth and subject to the mind as the mind’s mirror and reflection.”

Friedrich Nietzsche [1]

Scientific modelling is one of the best examples of a human activity fitting the words of Zarathustra: it requires the generation of conceptual representations for processes which frequently depend on uncomfortable features such as measurement inaccuracy, constituents interdependence or complex dynamics. We attempt to incorporate these representations within a mathematical or computational framework, which is nothing but a comfortable place where we reaffirm our confidence in the acquired knowledge. Building a formal framework provides a favourable environment to reach new analytical and computational results, thus accelerating the outcome of new predictions that can be firmly settled within the scientific knowledge after hypothesis testing.

Many interesting challenges in scientific modelling come from complex systems, which are commonly defined as systems composed of a large number of entities driven by non-linear interactions between their components and with the environment. In particular, complex systems may lead to a controversial phenomenon in modern science: the observation of emergent behaviours. Among this kind of collective behaviours we find phenomena such as magnetism, patterns observed in dissipative systems like hurricanes or convection cells or, in biological systems, patterns on animal skins or flocking behaviour. Looking at these examples it seems that the difficulty arises from an apparent discontinuity between the emergent macroscopic properties and their microscopic description. Since a basic tenet in the scientific method is that macroscopic properties are the consequence of the lower level constituents, a critical question arises here [2]: How is possible to obtain a satisfactory conceptual representation of emergent macroscopic behaviours when the definition of emergence apparently implies a discontinuity between the microscopic and the macroscopic representation?

Explaining the origin of this discontinuity has led to the famous controversy between the vitalist and reductionist approaches [3, 4]. Our first aim is not to discuss what an emergent property *is* or if it is possible to *generate* an emergent property—e.g. through a simulation or experiment. We rather wonder which are the features that a complex system exhibiting an emergent behaviour may have for being more or less accessible to an explanation of that behaviour by applying the scientific method. Nevertheless, the term *explanation* is central in the above discrepancy, as it is clearly apparent in this phrase summarizing the so called *emergentist* position, seen by Kim as a meeting point in the controversy: Emergent properties are identified when we observe that a complex system “begins to exhibit genuinely novel properties that *at a first sight* are irreducible to, and neither predictable nor explainable in terms of, the properties of their constituents” [5]. Although this statement brings a good intuition on when an emergent behaviour is identified, we added the words “at a first sight” to emphasize our discrepancy: although we agree in that emergent properties are not just a simple extrapolation of lower level properties –thus rejecting a simplistic compositional physicalism– this does not necessarily mean that emergent behaviours cannot be *explained* from a microscopic description and, why not, eventually *predicted*. At least as scientists, unless strong experimental and theoretical support reject this possibility, we prefer to remain agnostic. Following the view of Francis Crick, a natural starting point for a scientist is the decomposition of the system and we expect that “…its behaviour can, at least in principle, be understood from the nature and behaviour of its parts plus the knowledge of how all these parts interact” (reproduced from [4] referring to [6] p.11), i.e. this perspective admits an explanatory physicalism, and in the following we will try to precisely define what is understood here as *explanatory*.

**Fig 1:**
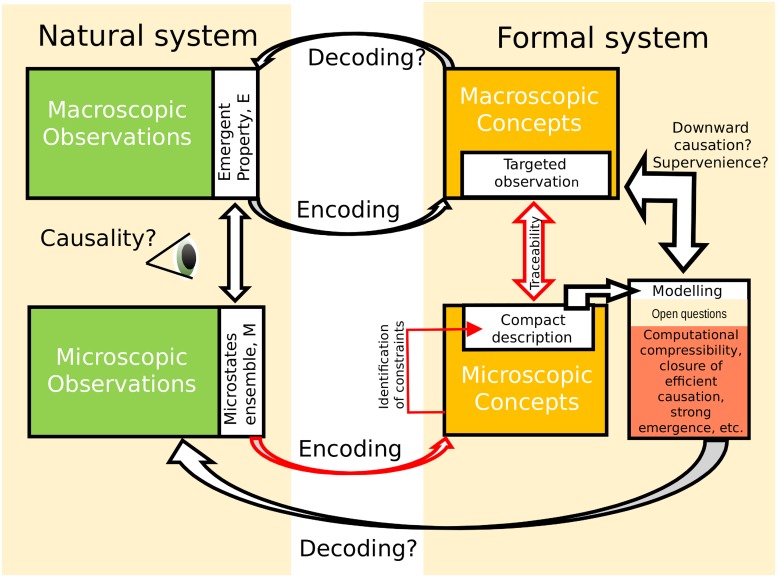
Scheme of the epistemological approximation of an observer in the analysis of emergent properties. Research starts with the observation of an emergent macroscopic property *E* and the associated ensemble of microstates *M*, both characterized through measurements. These observations are then encoded to build a formal framework. In this work, we are interested in finding a *minimal epistemological map* between the formal macroscopic and microscopic descriptions (red arrows). This map consists of finding a microscopic compact description of the ensemble, which is achieved through the identification of microscopic constraints in the dynamics of the system, in which case we will say that the macroscopic property is traceable. This is a necessary *a priori* step before a model is built (box modelling) that will allow the researcher to address other questions (see questions marks) such as supervenience of upper and lower level constituents or how to decode the formal framework under the scientific method through experiments. Interestingly, although we focus in a very specific epistemological process, and we do not explicitly address questions related with modelling or the ontology of emergent properties, the analysis of microscopic constraints sheds light over these questions.

In the above emergentist definition one should distinguish between *theoretical prediction* of the features of an emergent property *E* and its *inductive prediction* [5]. Inductive prediction occurs if, every time that the property *E* is observed, a particular ensemble of microstates *M* (see Fig 1) is also observed. The fact that this situation can be considered a phenomenological law justifies us to say that understanding what comprises the interrelations of the components of the system and the influence of the environment that leads to the observation of *M*, already constitutes an explanation of *E*. Still we may argue that this explanation is incomplete if we do not understand how visiting *M* translates into the appearance of *E*. Or if we are not able to *theoretically* predict the emergence of *E* when we operate over the system to make it run into *M*, formally through simulations or, in the natural side, decoding the formalism with experiments such that we intend to control the system to generate *M*, and hence to observe *E*. But we will defend here that it is the first step that should be tackled in the search for an explanatory framework of *E* under the scientific method.

Following this attitude, we wonder which are the properties that a complex system (and, in particular, those exhibiting emergent behaviours) may have for being more or less accessible to our knowledge by applying the scientific method, and we address this question by focusing on the analysis of the encoded description of *M*. In this sense, we align with the proposal of de Haan [7], who highlights the necessity of a general epistemological framework in which emergence can be addressed, and we need to incorporate in this framework arguments to determine what should be understood as epistemologically accessible.

An interesting condition for epistemological accessibility was proposed by Bedau when he coined the term *weak emergence* for those emergent processes that are epistemologically accessible only by simulation [8]. The idea is that a simulation would demonstrate the *supervenience* of upper level properties from lower level constituents, even if the mechanistic process leading to the observed pattern is not completely understood, i.e. it is not possible to compress the simulation into a compact set of rules explaining how the outcome is determined. Therefore, it provides an objective definition of emergence based on computational incompressibility, that has been explored by different models such as cellular automata [9, 10] or genetic algorithms [11], approximations that were later called *computational emergence* [12].

Nevertheless, Bedau also pointed out that if it is not possible from the simulations to recover regularities that may lead us to describe the system under compact laws, the above computational approximations should be understood as non-epistemic, hence being useless in deciphering the principles governing the emergence of a property [8]. Furthermore, Huneman rightly emphasized that this is an important question to understand the relationship between computational models and processes observed in nature [13] and, for those computational models depicting regularities in their global behaviour, coined the term *robustly emergent* models.

There is a last notion of emergence we would like to discuss that has been considered fundamental –as opposed to epistemological–, which is called *strong emergence* [14]. In physics it is frequently assumed that knowing the positions and velocities of particles is sufficient to determine the pairwise interactions. This assumption is frequently found in physics-inspired models of collective behaviour, where individual motion results from averaging the pairwise responses to each neighbour. Bar-Yam reasoned, however, that this assertion would not hold if the system is embedded in responsive media –such as the motions of impurities embedded in a solid– or in any process where global optimization (instead of local) is involved. In this way, if there is a constraint in the system acting on every component simultaneously and it is strong enough –i.e. a strong global constraint– it is not possible to determine the state of the system considering only pairwise interactions. In a sense, the parts are determined *downwards* from the state of the whole, with consciousness being a paradigmatic example suggested for strong emergence [15].

In this paper, we investigate which are the conditions that a natural system depicting an emergent property may possess to be compatible with the notion of robust emergence. To address this task, we will shift the attention from computational models to focus on the analysis of experimental data (see Fig 1). We will follow the view in which emergence is considered a “relation between descriptions of models of natural systems and not between properties of an objective reality in itself” [16] although, as we will see immediately, we will not renounce to talk about ontology. In particular, we aim to understand the relation between macroscopic descriptions of emergent behaviours and their microscopic counterparts, a map similar to the one proposed by Kim [5]. This map has been criticized by Mitchell saying that it is static, and that it cannot account for the dynamical nature of systems depicting emergent behaviours [17]. But here we show that the ability to find what we will call a *compact* description of the microstates’ ensemble depends indeed on the dynamical features of the system and, in particular, on how the components are entangled through internal and external constraints. In this way, the minimal explanation we look for resides in the description of the microstates’ constraints, and the epistemological accessibility will be related with our ability to find and compress such a description. Our strategy starts from experimentally characterized microscopic states associated to a macroscopic emergent observation, and investigates which kind of regularities found in these microstates are more difficult to compress and why. We believe that this is a necessary *a priori* step for any approach aiming to model the observed process, and thus scientifically sound notions of emergence should focus first on this process.

An immediate risk in this endeavour is that we must select a framework to work with and, by doing this, any result may depend on the framework selected. To circumvent this difficulty, we will apply constructive logic, which is both predicative and intuitionistic [18]. Since we aim to investigate the concept of emergence within a scientific setting, we will focus on how formal models are built starting from experimental measurements –that are considered basic characteristics– following a purely constructive attittude. This will allow us to monitor any loss of information [18], which we will see is particularly important to understand the difficulties in the formalization of emergence. In particular, the analysis of different synthetic examples with the rather limited repertoire of logical operations considered, will allow us to establish fair comparisons between systems, and to investigate further the ontological origins of these difficulties.

The article is articulated as follows. In Methods we introduce the formalism. We provide a careful definition of the system, and then introduce the different mathematical tools needed. Then we describe our epistemological approximation to the problem, explaining how a number of key concepts such as *constraint* or *model* should be understood within this framework. Then we explain the general procedure followed to analyse the systems, in which logical disjunction has a prominent role.

In Results, we analyse synthetic examples of 3-bit systems, to illustrate how the number and scope of the constraints hinder our ability to describe the systems. We next discuss the limitations of our approach, and how it is possible to elucidate which is the complexity of the constraints underlying the system under analysis despite these limitations, through the active *intervention* of the observer on the system. This procedure will lead us to propose a definition of emergence strength that we believe reconciles different conceptions of emergence found in the literature.

## Methods

### Main definitions and operations

#### System definition

We start by proposing a glossary of terms concerning the system definition, some of them close to those proposed by Ryan in [19]. We will call a set of basic quantities associated with a given entity an (object of) observation *o*_*i*_. Each of these quantities is a function *f* of the Cartesian product of a finite collection of sets –at least one of which is determined by experimental measurement– into real numbers R, i.e. f:A×B×...×P→R.

The non-measurable sets may refer to a set of measurement units (e.g. grams, meters), to a set of reference frameworks, or to any other set necessary to determine the final quantity. For simplicity, we will consider that any variation in magnitude is a consequence of a variation in the outcome of a measurement and thus, in the following, we will refer to measurements when the quantities describing objects of observation are discussed.

Hence, a system is characterized by a collection of *M* quantitative and/or qualitative (i.e. binary) magnitudes *X* = {*x*_*k*_, *k* = 1, .., *M*}. Given that we are interested in complex systems, we will consider that the system consists of a large number of entities, that we denote *N*. We will call this selection of objects *scope*, whose size, *N*, implicitly determines the spatio-temporal boundaries of the system. Determining the scope is already a difficult task for large complex systems. Difficulties arise, on the one hand, from the identification of these entities because, when their number is large, a complete characterization may be unfeasible. On the other hand, it will also be difficult to define the separation between system and environment, as oftentimes this separation cannot be achieved using strictly objective arguments [20, 21].

The selected variables *X* are intended to be sufficient to answer the questions addressed. For simplicity, we start by considering an ideal scenario in which all of these variables can be quantified for any entity within the system, leading to *N* × *M* specific values. In order to discuss how the complexity of a system can be explored using the scientific method, we will propose a procedure to relax this assumption in section.

Every variable *x*_*k*_ has a *precision* *r*_*k*_ which is its finest interval of variation and may be determined by different means. For instance, the precision may be limited by the intrinsic measurement error, which would be an ontological limitation. Another possibility arises when the expected influence of a given variable on the system’s description is small for a given shift in value, and a coarser discretization is then justified (a methodological choice). Calling *I*_*k*_ the interval of viable values of *x*_*k*_, the number of possible values considered for this variable will be *ζ*_*k*_ = *I*_*k*_/*r*_*k*_. Note that *ζ*_*k*_ can be seen as the resolution of the variable *x*_*k*_, and thus we will call resolution *R* of the system the finest description that allows us to distinguish two of its states *R* = max_*k*_({*ζ*_*k*_}) (*k* = 1, .., *M*).

This choice of variables together with the set of viable values will be called the *focus* *F* of the knowing subject, upper-bounded by *F* ∼ *M* × *R*. We finally call the set of specific values {*N*, *M*, *R*} the *scale*. A factor multiplying any of these values represents a change in the scale of the scope (if *N* is modified), or the focus (*M* or *R*). Note that, according to this definition, the scale is an ontological attribute as determined by *N*, but it also depends on epistemological ones, determined by *M* and *R*. Therefore, the breadth of the focus is heavily influenced by epistemological choices. Interestingly, it has been claimed that emergent behaviours (emergent theories) are the consequence of a change in the scope [19] (and not in the focus [22]).

#### Measurable properties and the definition of concepts

Let us start introducing some definitions, most of them already provided and justified in [23], that we recover here for completeness. For the sake of simplicity we will start considering that our objects of observation *o* ∈ *O* are the components of a complex system at a given time, i.e. we focus on a single microstate *μ* with *N* components, each of them described by *M* variables. For the moment, each of these components is what we consider an object of observation. We will move later towards a description where each object of observation is a microstate itself, becoming the whole space of objects the observed phase space. All the definitions considered in the following for a single microstate can be extended for other objects with a different scale.

**Definition: We call a *basic concept* or *characteristic*** $c_{a}=x_{k}^{*}$ **the specific value** $x_{k}^{*}$ **of a variable *x*_*k*_, out of the *ζ*_*k*_ possible values, measured over an object of observation** *o*. In this way, if we consider two different measurements for the same entity, each of them will constitute a different object of observation.

**Definition: We call *focus*** *F* **the whole set of characteristics considered by the observer**: $F=\left\{x_{k}^{l};\;k=1,..,M;\;l=1,..,\zeta_{k}\right\}\equiv \left\{c_{a};\;a=1,..,\tilde{M}\right\}$, **with** $\tilde{M}=\sum_{k=1}^{M}\zeta_{k}$. We make explicit here the discrete nature of the conceptual setting and the relation between resolution and focus, which achieves a suitable description in terms of characteristics, leading to the definition of concepts. Discreteness is ultimately a consequence of the non-vanishing systematic error associated with any measurement, and any transition into a continuous description would be a formal abstraction made during the modelling process.

**Definition: We call a *concept*** *ν* **any non-empty finite subset of** *F*: *ν* = {*c*_1_, …, *c*_*P*_}, **with** $P\leq \tilde{M}$. We defined the intension of concepts. Given that a concept may contain a single characteristic, *ν* = {*c*}, any characteristic can be considered a (basic) concept as well. Characteristics are atomic concepts in the sense that, for research purposes, they are not further decomposable into other concepts. In this sense, they constitute a basis for any other concept. The distinction between concept and characteristic will be needed in certain circumstances, but given the simplicity of the examples we discuss below, we will rarely use this distinction and refer simply to concepts.

#### Logical operations

It follows from the previous definitions the operations below to build new concepts.

**Definition: (Conjunction of concepts). Let** *ν*_1_ = {*c*_1_, …, *c*_*P*_} **and** *ν*_2_ = {*d*_1_, …, *d*_*Q*_} **be two concepts. Then, the conjunction of** *ν*_1_ **and** *ν*_2_ **is the concept**

$$\begin{array}{c} \nu_{1}\wedge \nu_{2}=\left\{c_{1},...,c_{P},d_{1},...,d_{Q}\right\} \end{array}$$ (1)

The conjunction of concepts is, in turn, a concept which consists of the set of all characteristics contained in concept *ν*_1_ AND *ν*_2_. Although the new concept is the union of two sets of concepts, note the choice of the meet symbol ∧ made in [23] that we also follow here. With this choice it is stressed that there are *fewer* concepts enjoying all the characteristics in the union than concepts enjoying all the characteristics of the finite sets *ν*_1_ and *ν*_2_ separately (Silvio Valentini, personal communication). The situation is confirmed by the choice of the empty set as the greatest element among collections of characteristics, given that the characteristics it contains (namely, no characteristics) are enjoyed by all concepts.

Now we would like to know the supremum of two concepts, namely the subset of concepts *U* that allows us to speak of the concept *ν*_1_ OR *ν*_2_.

**Definition: (Disjunction of concepts). Let** *ν*_1_ = {*c*_1_, …, *c*_*P*_} **and** *ν*_2_ = {*d*_1_, …, *d*_*Q*_} **be two concepts. Then, the disjunction of** *ν*_1_ **and** *ν*_2_ **is the subset**

$$\begin{array}{c} U=\nu_{1}\vee \nu_{2}=\left\{\nu_{1},\nu_{2}\right\}=\left\{\left\{c_{1},...,c_{P}\right\},\left\{d_{1},...,d_{Q}\right\}\right\} \end{array}$$ (2)

This operation is a sensible one because we will use it to describe subsets of observations such as {*ν*_1_, *ν*_2_}, and we would like to find a concept for this subset describing either the observation of *ν*_1_ OR the observation of *ν*_2_ OR the observation of *ν*_1_ AND *ν*_2_. Unfortunately, this concept does not generally exist in a closed form, a fact that will be central to our investigation.

The space of concepts described so far, equipped with a join and a meet for every finite subset, fulfils the properties of a distributive lattice [24] (moreover, it is a topology [25], although we will not exploit this fact) and hence we can get some more intuition about disjunction through the following property.

**(Distributivity of the disjunction of concepts). Let** *ν*_1_ = {*c*_1_, …, *c*_*P*_, *b*_1_, …, *b*_*L*_} **and** *ν*_2_ = {*d*_1_, …, *d*_*Q*_, *b*_1_, …, *b*_*L*_} **be two concepts. Then the subset of concepts** *U* = *ν*_1_ ∨ *ν*_2_ **can be expressed as**

$$\begin{array}{c} U=\nu_{1}\vee \nu_{2}=\left\{b_{1},...,b_{L}\right\}\wedge \left[\left\{c_{1},...,c_{P}\right\}\vee \left\{d_{1},...,d_{Q}\right\}\right]. \end{array}$$ (3)

Therefore, disjunction of concepts leads to a subset of concepts containing the set of all characteristics in common to both concepts, plus those that are observed either in one or the other. As we already noted, we are unable to build a concept, let us say *q*, in terms of conjunctions of concepts to talk about *U*, but it is clear that the more concepts *ν*_1_ and *ν*_2_ have in common the closer we are to finding such a concept. Other basic properties of conjunction and disjunction that will be used are the following [24]:

$$\begin{array}{ccc} & \wedge & \vee \\ Commutativity & \nu_{1}\wedge \nu_{2}=\nu_{2}\wedge \nu_{1} & \nu_{1}\vee \nu_{2}=\nu_{2}\vee \nu_{1}\\ Associativity & \left(\nu_{1}\wedge \nu_{2}\right)\wedge \nu_{3}=\nu_{1}\wedge \left(\nu_{2}\wedge \nu_{3}\right) & \left(\nu_{1}\vee \nu_{2}\right)\vee \nu_{3}=\nu_{1}\vee \left(\nu_{2}\vee \nu_{3}\right)\\ Unit\;laws & \nu_{1}\wedge \text{true}=\nu_{1} & \nu_{1}\vee \text{false}=\nu_{1}\\ Idempotence & \nu_{1}\wedge \nu_{1}=\nu_{1} & \nu_{1}\vee \nu_{1}=\nu_{1}\\ Absorption & \nu_{1}\wedge \left(\nu_{1}\vee \nu_{2}\right)=\nu_{1} & \nu_{1}\vee \left(\nu_{1}\wedge \nu_{2}\right)=\nu_{1} \end{array}$$

#### The space of objects of observation

We have introduced a *formal* conceptual apparatus that a knowledge subject may use to build from experimental measurements a model, but we still do not know what a model means. Before getting into this discussion, we should establish a map between the formal side in which we developed our framework and the *concrete* space, in which the objects of observation live, i.e. the objects that constitute the physical world. In particular, we are interested in understanding how the set of concepts we considered, which determines a partition of the focus, will induce, in turn, a partition in the set of objects of observation. In other words, we aim to determine a constitutive relationship between any single characteristic belonging to the focus *F* and the set of objects *O*. Understanding this map will allow us to work in the formal space while ignoring the concrete space, but being certain that any new concept arising in the formal side has a correspondence in the concrete side. The following constitutive relationship will express how the objects become cognitively significant by means of the characteristics measured and, in turn, by the concepts we build from them [23].

**Definition: (Constitution relation). Let** *F* **be the focus over a set** *O* **of objects. Given** *o* ∈ *O* **and** *ν* ∈ *F*, **we introduce a binary relation**, ⊩, **that we call *constitution relation*, such that by** o ⊩ ν **we mean that *ν* is one of the concepts constituting** *o*. With the constitution relation we determine how the objects of observation are expressed via the conceptual apparatus of the knowing subject. In addition, we would like to know which objects are constituted by a given concept.

**Definition: (Extension of a concept). Let** *ν* ∈ *F* **be a concept. Then, the extension** Ext **of** *ν* **is the subset of objects of** *O* **constituted by** *ν*, **that is**

$$\begin{array}{c} \text{Ext}(\nu )=\{o\in O\;|\;o⊩\nu \} \end{array}$$ (4)

We note here that an immediate consequence of Eq 4 is that any object of observation has necessarily associated with it a concept, i.e. it is just cognitively accessible by means of the conceptual apparatus of the knowing subject. This assertion, if accepted in general, leads to a Kantian epistemological positioning [25]. In our case, it is a consequence of the fact that our objects of observation are built from measurements of a reproducible experimental setting, and hence it is true by construction. Nevertheless, the opposite is not true, as we may deal with concepts for which no object is observed, i.e. Ext(*ν*) = ∅. Such a situation would arise for example if we have *a priori* expectations of the viable values of the system. For instance, we know that a group of birds can fly in any direction even if we systematically observe them flying in a single direction. From the point of view of the scientific method, concepts built from *a priori* expectations are very important, as they may be used to propose null hypotheses which, in general, can be formulated as *H*_0_: "*ν* observed". It is when we reject the hypothesis through experiments that we acquire a scientific knowledge of the process analysed, i.e. that *ν* is not observed with some statistical significance.

Finally, we aim to know what is the extension of a subset *U* of concepts *U* = {{*ν*_1_}, …, {*ν*_*L*_}}.

**Lemma: Let** *U* **be a subset of the set** *F* **of concepts. Then, the extension of** *U* **is defined by setting**

$$\begin{array}{c} \text{Ext}\left(U\right)=\underset{\nu \in U}{\cup }\text{Ext}\left(\nu \right) \end{array}$$ (5)

Hence, if we consider two concepts *ν*_1_ and *ν*_2_, we should not confuse the extension of a concept built by conjunction of concepts *ν* = *ν*_1_ ∧ *ν*_2_ = {*c*_1_, …, *c*_*P*_} ∧ {*d*_1_, …, *d*_*Q*_} = {*c*_1_, …, *c*_*P*_, *d*_1_, …, *d*_*Q*_} with the extension of a subset of concepts built by disjunction *U* = {*ν*_1_, *ν*_2_} = *ν*_1_ ∨ *ν*_2_ = {{*c*_1_, …, *c*_*P*_}, {*d*_1_, …, *d*_*Q*_}}. In the former case, we look for objects containing both concepts and, thus, the number of these objects is smaller than or equal to the number of objects described by *ν*_1_ or *ν*_2_, Ext(*ν*) = *Ext*(*ν*_1_ ∧ *ν*_2_) = *Ext*(*ν*_1_) ∩ *Ext*(*ν*_2_). On the other hand, the subset *U* extends over objects containing *any* of the concepts, since its extension is the union of the extension of both concepts, as shown in Eq 5.

### Epistemological approximation

#### Microscopic and macroscopic descriptions

Following the definitions introduced, we would like now to differentiate between two types of variables providing a description of the system at different scales: microscopic and macroscopic variables. Note that by microscopic we do not mean “atomistic” but instead a significantly shorter spatio-temporal scale of observation of the system. A particular feature of the interplay between both scales is that, when a macroscopic property is observed during the dynamical evolution of a system, even if the microscopic variables are continuously changing, the macroscopic variable’s values remain the same.

In the following, we will call microstate *μ* a vector containing, at a given time, the values of a set of variables {*x*_*k*_} that fully determines the state of the microscopic objects, i.e. $\mu =\{x_{k}^{*}\}$, where $x_{k}^{*}$ stands for a particular value of the variable *x*_*k*_. Therefore, the basic objects of observation we are considering now are the microstates *o* ≡ *μ*. When a coarse-graining of the microstates at the spatial, temporal, or spatiotemporal dimensions is performed, it may be possible to determine macroscopic variables *y*_*k*_ describing the state of the system at the new (coarser) scale. We will call macrostates the objects described at the macro scale *o* ≡ *ξ*. In some cases, the macroscopic variables *y*_*k*_ can be obtained by applying a surjective map *f* over the microscopic variables *f*(*x*_*k*_)→*y*_*k*_. For instance, if we deal with an incomplete (statistical) microscopic description of an ensemble, *P*(*μ*), we can obtain a coarse determination of a macroscopic variable *y*_*k*_ averaging the correspondent microscopic variable *x*_*k*_, weighted by the statistical probability of the microstates over the ensemble 〈*x*_*k*_(*μ*)*P*(*μ*)〉. Nevertheless, in many other situations it is not possible to find such a map, and we argue that this fact underlies many problems surrounding the study of emergent properties: we observe a macroscopic property such as the collective behaviour of many interacting elements, and it does not seem possible to explain it from lower levels of description (for instance, from the properties of the entities themselves).

It is important to underline that macrostate and microstate definitions are relative to the scale of observation and they may change if we move from one scale of description to another. Consider a system described within a certain temporal scale by a set of microstates {*μ*_*i*_} which are associated with the observation of a single macrostate *ξ*. Assume now that the system evolves along a sufficiently long path such that we observe different macrostates and we store *T* snapshots of these dynamics, leading to an ensemble of macrostates ${\left\{\xi_{u}\right\}}_{u=1}^{T}$. If at this larger scale new properties arise, we may be interested in considering that each of these macrostates is now a microstate $\hat{\mu }$ for a new system with a larger scope and lower resolution $\xi_{u}\to {\hat{\mu }}_{i}$. Given that the scope of a macrostate will always be larger than that of a microstate (*N*_*ξ*_ ≥ *N*_*μ*_), whereas the opposite is true for the resolution (*R*_*ξ*_ ≤ *R*_*μ*_), in this exercise we have increased the scope and reduced the resolution. This is the reason why, the larger the scale, the more difficult it is to build a bottom-up explanatory framework.

We should also note that this change in scale requires an effort to reduce the system description, but this kind of reduction has been performed from the very first step: for the definition of scope, we have neglected entities; for the definition of focus, we have neglected variables and probably restricted their viable values assuming a lower resolution. Furthermore, any map between microstates and macrostates again considers a reduction in the information provided by the microstates. In general, for both very broad or very detailed questions the technical complexity increases and a reduction in description is unavoidable, and it is important to remark that this exercise is not linked with a *naive* reductionist positioning, in which it is accepted that any macroscopic description is a simple extrapolation of the properties of the microscopic description [26]. Instead, we accept that in complex systems there are discontinuities between the different levels of description and that, for each new level, new properties may arise. We are interested here in investigating when a microscopic description is a minimal representation of an emergent macroscopic observation.

#### Traceability, compact descriptions and models

Given the above considerations, we propose two formal definitions that will be helpful for understanding our rationale behind the further development of the paper. Before getting into the first definition, we should clarify what is understood as constraints of the system, which are considered here simply as restrictions in the viable values of the variables we handle [27]. Any system is constrained to some extent. But there are some constraints that belong to the definition of the system itself, that we will call *intrinsic*, and others that depend on particular conditions, that we will call *facultative*. Consider the structural differences between a protein and a heteropolymer whose sequence is the result of random shuffling of the protein sequence. Both chains of amino acids have the same number of intrinsic constraints (those derived from the existence of peptidic bonds), but a protein structure requires three additional constraint levels in the interactions between its amino acids that allow the polymer to behave as a protein: the first is needed for it to be kinetically foldable in a biologically relevant time, the second for making the fold thermodynamically stable under physiological conditions, and the third to perform its specific function (metal-binding, phosphorilation, etc.). Both chains have the same amino acids but evolution has selected for a specific order in the sequence that generates the constraints needed for the emergent property (the protein function) to arise. Quantitatively, the probability that these constraints should appear by chance is quite low: the number of possible heteropolymers of length *N*, considering an alphabet of 20 amino acids, is 20^*N*^ and, as a reference, the number of protein structures (of *any* length) deposited to date in the Protein Data Bank (although far from being complete) is only ∼1.2 × 10^5^ (www.pdb.org).

Calling {*μ*_E_} the set of microstates visited when an emergent behaviour is observed, {*μ*_NE_} the set of microstates not visited, and *Ω* the whole phase space, we expect that #(Ω) ≈ #({*μ*_NE_}) ≫ # ({*μ*_E_}); where #({⋅}) is the cardinality of the set {⋅}, i.e. the number of elements it contains. Therefore, we expect the volume of the region of the phase space where an emergent property arises to be much smaller than the whole phase space. This is probably why unpredictability or surprise are attributes frequently used to describe emergent properties.

Let us now define what we consider a macroscopic property, whose identification is typically the starting point of any research.

**Definition: (Target macroscopic property). We will say that an observed macroscopic property is a target (of scientific research) if it is only observed when certain microscopic facultative constraints are present**. With this definition we state a minimal condition for considering that a macroscopic property is susceptible of being analysed through the scientific method, an assumption that also applies when an emergent property is interrogated. Therefore, although it is an epistemological condition, it implies an ontological assumption about emergent properties, namely that these are the consequence of a microscopic behaviour running under facultative constraints.

Hence, we consider situations in which the phase space of the system Ω is restricted to a smaller observed region Ω^O^ ⊂ Ω, and we say that there exists a (perhaps novel) macroscopic concept $\hat{c}$ such that $\text{Ext}\left(\hat{c}\right)=\Omega^{O}$. This fact motivates the exploration of this region both in terms of macroscopic $\left\{\hat{c}\right\}$ and microscopic {*c*} concepts (see Fig 1). We now introduce a condition that allows us to consider that a macroscopic description is in correspondence with a microscopic description.

**Definition: (Traceability). Given a target macroscopic property** $\hat{c}$ **and the observed phase space** Ω^O^ **associated with that property, i.e**. $\text{Ext}\left(\hat{c}\right)=\Omega^{O}$, **we will say that the macroscopic description obtained is traceable if we find an appropriate function or algorithm applied to microscopic properties** *f*: {*c*}→*q* **such that the new concept** *q* **derived *compactly* describes the ensemble of microstates, i.e**. $\text{Ext}\left(q\right)=\text{Ext}\left(\hat{c}\right)=\Omega^{O}$. This definition paves the way for quantifying the correspondence between both descriptions within the proposed framework. Note it does not mean that we should be able to thoroughly *explain* the macroscopic properties from the microscopic properties. It is a weaker condition that only requires establishing a correspondence between microscopic and macroscopic variables describing the same region of the observed phase space Ω^O^. In Fig 1 we make explicit this distinction showing how traceability appears as a relation between representations (double red arrows), while investigating causal relationships requires us to build models from the starting descriptions and to perform experiments (white arrow to modelling box). Therefore, traceability can be seen as a rather minimal epistemological condition that allows us to talk about emergent properties circumventing any discontinuity between both descriptions.

Let us think further about the existence of concepts *q* in the context of complex systems. After identifying a set of microstates *μ* visited when an emergent property is observed, we may be able to characterize this set as described above, through a set of concepts {*e*_*i*_ / Ext(*e*_*i*_) = *μ*_*i*_, ∀*i*}. Given the intrinsic dynamical nature of these systems, a natural starting point in the search for a formal description will be given by *q* = *e*_1_ ∨ … ∨ *e*_*L*_, *L* being the number of microstates. Note, however, that the concept *q* simply presents the microstates but it does not provide any insight into the mechanistic processes underlying the observation of these microstates and no others. In the remainder of the article, we will show that to provide such mechanistic understanding it is necessary for *q* to constitute a description of the constraints of the system. Furthermore, the chances of building a mechanistic model will be related with our ability to build a compact description.

**Definition: (Compact description) We say that a set of distinguishable microstates** {*μ*}, **namely a set in which every microstate** *μ*_*i*_ **is unambiguously described by a concept** *e*_*i*_ ∈ *F*, *F* **being the microscopic focus, is compactly described by a concept** *q*, **if** Ext(*q*) = {*μ*} **and it *irreversibly* describes the constraints existing in the system**. By irreversibility it is meant here that, even if *q* is analytically derived from the starting concepts *e*_*i*_ and it is still a definition of the system, and thus Ext(*q*) = *Ext*({*e*}), we lose information in the derivation in such a way that it is impossible to reverse the operations and retrieve back the original states. To recover the full description of the system it will be needed to create a model.

**Definition: (Model) A computable function or algorithm is a model** Ψ **if, given a compact description** *q* **of a set of microstates** {*μ*}, **it interprets the constraints encoded in** *q* **and generates the description of the microstates from which the compact description was derived, i.e**. Ψ(*q*) = {*e*}. In summary, we are interested in investigating the traceability of complex systems through compact descriptions describing the constraints of the system, which may allow us to build mechanistic models to test hypotheses about the accuracy of the description found. Note that, if the description of the constraints is incomplete or incorrect, a model will generate a set of microstates that would not perfectly match the observed microstates (if it is able to match them at all), a fact that will be exploited in our proposal to quantify emergence.

The process we describe in the following is similar in spirit to Rosen’s attitude when he said that, in the modelling process, instead of throwing away the organization and keeping the matter (a compositional physicalism) one should throw away the matter and keep the organization (see Chap 5E in [28]). While looking for constraints we shed light on the entailment between the components of the system. Still, to prove that the constraints found correspond with the observed microscopic process, we should use a generative model to show that the original microstates are recovered. The condition of irreversibility tries to avoid an impredicative definition of the system’s constraints: we aim to avoid any definition of the system’s constraints that consists of the mere presentation of the system itself.

## Results

### Identification of constraints: Focusing on disjunction

As we anticipated, when an emergent property is observed the probability distribution of the values of one or several variables depart from the distribution observed when the system is free of constraints, thus losing ergodicity [29] (p. 186). Therefore, the existence of facultative external or internal constraints limit the behaviour of the system and, as we will attempt to clarify, a necessary condition to determine a microscopic property associated to every microstate visited requires the determination of the existing constraints. Indeed, it has been claimed that the reduction in the degrees of freedom of the system –a phenomenon for which it has been coined the term dissolvence– relies at the basis of the formation of complex emergent structures [30]. We will show here that the nature of the different constraints acting on the system determine its epistemological accessibility, and hence our ability to reach a satisfactory explanation of emergent behaviours.

Firstly, we show how the extension of those concepts built through binary operations over sets of concepts can be obtained. Given a new concept *α* built via conjunction of two concepts, *ν*_1_ and *ν*_2_, i.e. *α* = *ν*_1_ ∧ *ν*_2_, its extension is Ext(*α*) = *Ext*(*ν*_1_) ∩ *Ext*(*ν*_2_). With conjunction we reduce the scope of the description but we refine it, being therefore the basic operation needed to build sharp descriptions of the observed objects.

**Fig 2:**
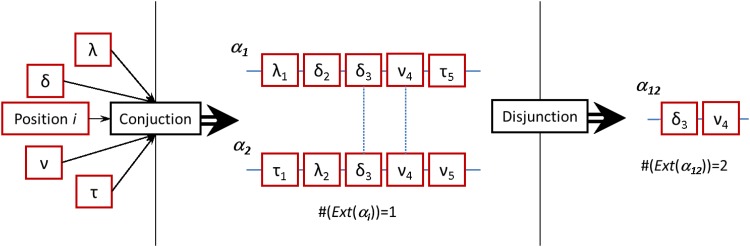
Illustration of conjunction and disjunction of concepts. Starting from the knowing subject’s conceptual apparatus (Greek letters, left), two sequences *α*_1_ and *α*_2_ are built through conjunction of the basic concepts, being themselves concepts (center). These sequences uniquely determine single objects, for instance protein sequences, and thus #(Ext(*α*_*i*_)) = 1. Comparing both sequences we observe two common concepts (linked by dotted lines) that we identify through binary disjunction. If these two concepts are only found at these sequences, we can say that a new concept *α*_12_ sharply describes these sequences (right). This concept contains less basic concepts, but the extension is larger than the original sequences, i.e. #(Ext(*α*_12_)) = 2.

Let us take as an example the description of polymers of amino-acids {*o*_*α*_}. Each amino-acid is described by two quantities that we will consider basic characteristics: the position in the sequence and its specific amino-acid identity. In our framework, an example of a concept describing an amino-acid is *ν*_*i*_ = “cysteine in position *i*” which, in turn, is built by conjunction of the characteristics “*cysteine*” and “*i*”. The whole polymer *o*_*α*_ will be described by a concept *α*, built by conjunction of a set of concepts *ν*_*i*_, i.e *α* = (λ_1_ ∧ *τ*_2_ ∧ … ∧ *ν*_*N*_) (see Fig 2). The sequence becomes uniquely determined with the concept *α* because its extension exactly maps the polymer under study: Ext(λ_1_ ∧ *τ*_2_ ∧ … ∧ *ν*_*N*_) = *Ext*(*α*) = *o*_*α*_. In summary, conjunction underlies bottom-up approximations, for which we look for sharp descriptions adding up concepts.

Let’s now consider disjunction. Given two polymer sequences *α*_1_ and *α*_2_ (see Fig 2), described as *α*_1_ = (λ_1_ ∧ *δ*_2_ ∧ *δ*_3_ ∧ *ν*_4_ ∧ *τ*_5_) and *α*_2_ = (*τ*_1_ ∧ λ_2_ ∧ *δ*_3_ ∧ *ν*_4_ ∧ *ν*_5_), we would like to look for a concept *α*_12_ that sharply describes both sequences and no others. Intuitively, such a concept might be found if we can build a concept through conjunction of those concepts shared by both sequences. Following the example, by using Eq 3 we obtain:

$$\begin{array}{c} \alpha_{1}\vee \alpha_{2}=\left(\delta_{3}\wedge \nu_{4}\right)\wedge \left[\left(\lambda_{1}\wedge \delta_{2}\wedge \tau_{5}\right)\vee \left(\tau_{1}\wedge \lambda_{2}\wedge \nu_{5}\right)\right], \end{array}$$ (6)

what it immediately highlights is that there is a candidate for a common concept describing both sequences, *α*_12_ = *δ*_3_ ∧ *ν*_4_ (see Fig 2). However, finding a concept describing common information is a necessary but not sufficient condition, because we should also guarantee that *α*_12_ is only found in these sequences in order to safely separate them from any other, what is not true in general.

**Fig 3:**
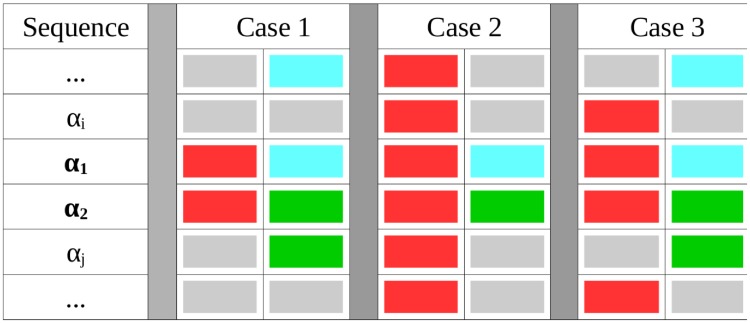
Illustration of disjunction of two concepts. We consider two concepts *α*_1_ and *α*_2_, sharply defined by conjunction of red (*ν*_1_ in the Main Text) and cyan (*ν*_2_) for *α*_1_, and by conjunction of red and green (*ν*_3_) for *α*_2_. The grey concepts denote any other colour needed to sharply determine the other *α*_*i*_ concepts. Since these three colours suffice to sharply describe *α*_1_ and *α*_2_, their extensions over the different objects of observation must follow one of the three general cases shown in the figure and described in the Main Text.

In which situations can be guaranteed that a concept describing a set of objects exists? We can get some intuition with a simple example. Let’s consider two objects *o*_1_ and *o*_2_ sharply described by the concepts *α*_1_ = *ν*_1_ ∧ *ν*_2_ and *α*_2_ = *ν*_1_ ∧ *ν*_3_, meaning that Ext(*α*_1_) = *o*_1_ and Ext(*α*_2_) = *o*_2_. Distributivity of disjunction leads to

$$\begin{array}{c} \alpha_{1}\vee \alpha_{2}=\nu_{1}\wedge \left(\nu_{2}\vee \nu_{3}\right). \end{array}$$ (7)

which tell us that, to separate these objects from any other, a limited number of situations, illustrated in Fig 3, can occur:

1. Case 1. If Ext(*α*_1_) ⊆ *Ext*(*ν*_2_) and Ext(*α*_2_) ⊆ *Ext*(*ν*_3_), then, necessarily Ext(*ν*_1_) = *Ext*(*α*_1_ ∨ *α*_2_) and *α*_12_ = *ν*_1_ constitutes a sharp description of both concepts.
2. Case 2. If Ext(*α*_1_ ∨ *α*_2_) ⊂ *Ext*(*ν*_1_) then Ext(*α*_1_) = *Ext*(*ν*_2_) and Ext(*α*_2_) = *Ext*(*ν*_3_) must hold, what leads to define the concept *α*_12_ = *ν*_2_ ∨ *ν*_3_.
3. Case 3. If Ext(*α*_1_) ≠ *Ext*(*ν*_2_) and Ext(*α*_2_) ≠ *Ext*(*ν*_3_), and Ext(*α*_1_ ∨ *α*_2_) ⊂ *Ext*(*ν*_1_) and we can only consider the possibility that *α*_12_ = *ν*_1_ ∧ (*ν*_2_ ∨ *ν*_3_) to be a sharp description.

Independently of the situation found, with disjunction we are able to observe the constraints existing in the system. In the first two examples we can talk about the two sequences saying that are those with *ν*_1_ in the first position (first case) or with either *ν*_2_ or *ν*_3_ in the second position (second case), highlighting the restrictions to other values. The third example, which is a combination of both, requires more information to be expressed.

We argue that, in complex systems, the third situation is rather the rule because variables are interlinked through internal and external constraints. And we could expect that the larger the number of objects and properties are, the more complex the expressions are. Under some circumstances, too complex expressions might reflect an imperfect state of knowledge that do not allow us to find more compact descriptions of the system. To avoid this situation, we will deal with a system in which the whole phase space is known in the next sections, and we will show in this way that it is the number and scope of the constraints what makes the description of the system’s constraints more or less complex.

### A synthetic example: The three bits system

We are already equipped with the necessary tools to analyse a synthetic example in detail. The toy model we consider consists of a system of three entities whose physical states are described by a single binary variable, i.e. a system modelled with three bits. Examples of this kind of systems may be sets of genes that are expressed (silent) when the amount of the correspondent protein is above (below) a certain threshold, species that are observed (absent) in certain environmental sample or the attractor of a boolean network. Each measurement performed over these entities will be considered an observation, taking a value of one or zero. Note that variable distiguishability is relevant, but the order in which the information is collected is unimportant. The reason is that these are not time-ordered strings but rather states of a system, and we assume that a state does not change within the scale of time needed to characterize it through measurements.

For a system composed by three binary entities the focus is

- *c*_1_ = ′ *ON* *at* *object* 1′; *d*_1_ = ′ *OFF* *at* *object* 1′;
- *c*_2_ = ′ *ON* *at* *object* 2′; *d*_2_ = ′ *OFF* *at* *object* 2′;
- *c*_3_ = ′ *ON* *at* *object* 3′; *d*_3_ = ′ *OFF* *at* *object* 3′;

and, with this focus, we can potentially observe 2^3^ = 8 microstates *μ*_*k*_ = (*x*_1_, *x*_2_, *x*_3_) (with *k* = 1, .., 8; and *x*_*i*_ = {0, 1}; see Table 1).

**Table 1:** Microstates of a 3-bit system.

Microstate	
*μ*_1_ = (1, 1, 1)	*μ*_5_ = (0, 1, 1)
*μ*_2_ = (1, 0, 0)	*μ*_6_ = (1, 0, 1)
*μ*_3_ = (0, 1, 0)	*μ*_7_ = (1, 1, 0)
*μ*_4_ = (0, 0, 1)	*μ*_8_ = (0, 0, 0)

Each microstate is defined in terms of this focus through concepts *e*_*k*_ (*k* = 1, …, 8) built by conjunction of characteristics. For instance, the microstate *μ*_7_ = (1, 1, 0) is defined in terms of the basic characteristics as *e*_7_ = *c*_1_ ∧ *c*_2_ ∧ *d*_3_, in turn being a concept. We can define for the 3-bit system (Nnext) possible combinations of constraints involving *n*_ext_ variables, and thus the number of final microstates will depend on the number of constraints and on their scope, i.e. the number of elements influenced by the constraint. In the following, we consider examples with a different number and type of constraints, all resulting in the same number of microstates (four out of the eight viable states). Hence, these constraints are codified in one bit of information, but we will see that the number of concepts needed to express these constraints can change from system to system.

The simplest macroscopic description associated to the observed ensemble arises if we consider a coarse graining of the microscopic properties such that there is a surjective map between microstates and macrostates: a macroscopic variable takes the value ′*ON*′ if these microstates are visited and ′*OFF*′ otherwise. In this way, only if there is a statistically significant bias towards these microstates we can say that a novel target emergent macroscopic property is observed.

Taking these considerations in mind, we aim to disentangle the microscopic constraints in the system following the present formalism. Given that we build our conceptual setting starting from the basic characteristics (obtained from measurements) and then performing binary logical operations, we expect that the results obtained for the different systems are fairly comparable.

#### System with a single constraint of scope one (S1)

The rational is the same for the three systems introduced. We consider that there is an observed macroscopic emergent observation ${\hat{c}}_{E}$, and we know the microstates belonging to the associated region of the phase space {*μ*_E_}. Then, we analyze the set of microstates looking for its constraints.

The first system we consider is a system where the first bit is constrained to a fixed value (*c*_1_), leading to the observations {*μ*_1_, *μ*_2_, *μ*_6_, *μ*_7_} that we explicitly show in Table 2.

**Table 2:** 3-bit microstates of a system with a single constraint of scope one.

{*μ*_E_}
*μ*_1_ = (1, 1, 1)
*μ*_2_ = (1, 0, 0)
*μ*_6_ = (1, 0, 1)
*μ*_7_ = (1, 1, 0)

In order to find the system’s constraints we start presenting the ensemble through disjunction of the concepts *e*_*i*_, which sharply describe the different microstates, and then we investigate if it is possible to find a compact description. We first expand the definition of the system:

$$\begin{array}{c} \Omega =e_{1}\vee e_{2}\vee e_{6}\vee e_{7}=\left(c_{1}\wedge c_{2}\wedge c_{3}\right)\vee \left(c_{1}\wedge d_{2}\wedge d_{3}\right)\vee \left(c_{1}\wedge d_{2}\wedge c_{3}\right)\vee \left(c_{1}\wedge c_{2}\wedge d_{3}\right) \end{array}$$

For clarity, we start looking for a compact description analysing the first two terms:

$$\begin{array}{ccc} e_{1}\vee e_{2}=\left(c_{1}\wedge c_{2}\wedge c_{3}\right)\vee \left(c_{1}\wedge d_{2}\wedge d_{3}\right) & = & c_{1}\wedge \left(c_{1}\vee d_{2}\right)\wedge \left(c_{1}\vee d_{3}\right)\\ & & \wedge \left(c_{2}\vee c_{1}\right)\wedge \left(c_{2}\vee d_{2}\right)\wedge \left(c_{2}\vee d_{3}\right)\\ & & \wedge \left(c_{3}\vee c_{1}\right)\wedge \left(c_{3}\vee d_{2}\right)\wedge \left(c_{3}\vee d_{3}\right)\\ & = & c_{1}\wedge \left(c_{3}\vee c_{2}\right)\wedge \left(d_{2}\vee d_{3}\right)\\ & = & c_{1}. \end{array}$$

In the simplification, *c*_1_ absorbed several terms and expressions of the type (*c*_*i*_ ∨ *d*_*i*_) or (*c*_3_ ∨ *c*_2_) ∧ (*d*_2_ ∨ *d*_3_) are mutually exclusive, hence vanishing. We proceed now similarly to simplify *e*_6_ ∨ *e*_7_:

$$\begin{array}{ccc} e_{6}\vee e_{7}=\left(c_{1}\wedge d_{2}\wedge c_{3}\right)\vee \left(c_{1}\wedge c_{2}\wedge d_{3}\right) & = & c_{1}\wedge \left(c_{1}\vee c_{2}\right)\wedge \left(c_{1}\vee d_{3}\right)\\ & & \wedge \left(d_{2}\vee c_{1}\right)\wedge \left(d_{2}\vee c_{2}\right)\wedge \left(d_{2}\vee d_{3}\right)\\ & & \wedge \left(c_{3}\vee c_{1}\right)\wedge \left(c_{3}\vee c_{2}\right)\wedge \left(c_{3}\vee d_{3}\right)\\ & = & c_{1}\wedge \left(c_{2}\vee c_{3}\right)\wedge \left(d_{2}\vee d_{3}\right)\\ & = & c_{1}. \end{array}$$

Combining both expressions yields

$$\begin{array}{ccc} \Omega & = & e_{v1}\vee e_{2}\vee e_{6}\vee e_{7}=c_{1} \end{array}$$

The result highlights that the system has a single constraint over the first variable, that does not allow us to observe the value *d*_1_, keeping the other two variables free. And the cost of this reduction is that we have irreversibly lost all the information needed to analytically recover the original concepts describing the system: a model would be needed to interpret this constraint and generate back these states.

**Fig 4:**
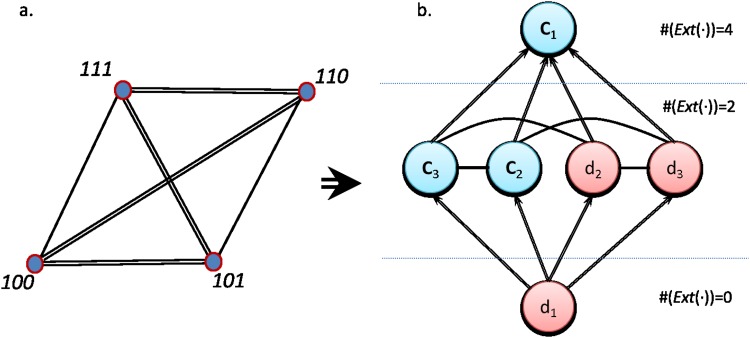
Representations of a three bits system with a single constraint of scope one. (Left) In the concrete network, each node represents a microstate and it is linked with another microstate if they share the same observation for any component, where the number of links represent the number of concepts shared. (Right) Formal network of concepts extracted from the analysis of the microstates. Two links *c*_*i*_ and *c*_*j*_ are linked with a directed edge if Ext(*c*_*i*_) ⊆ *Ext*(*c*_*j*_) and with an undirected link if Ext(*c_i_*) ∩ Ext(*c_j_*) ≠ ∅. The concepts are hierarchically ordered according to the cardinality of their extension, i.e. the number of microstates they map. In this example, a single constraint on *x*_1_ naturally arises, as one of its possible values maps the empty set.

To illustrate the relational rather than compositional nature of the approximation, we propose two representations that further allow us to see the interplay between the formal result found and the concrete representation of objects (see Fig 4). The first network represents the concrete side, where each microstate *μ*_*i*_ is linked with another *μ*_*j*_ if the same value is observed in the same variable. The constraints determine the microstates acting on the variables, so we should still move to the formal side to identify them. We create a second network considering now basic concepts as nodes, linking two concepts *c*_*i*_ or *d*_*i*_ if they extend over the same microstates (see Fig 4). More precisely, we link two concepts *c*_*i*_ and *c*_*j*_ with a directed edge if Ext(*c*_*i*_) ⊆ *Ext*(*c*_*j*_), and with an undirected edge if Ext(*c*_*i*_)∩*Ext*(*c*_*j*_) ≠ ∅. In this way, we compactly represent all the dependencies present in the system with relationships of subordination (directed edges) cooccurrence (undirected) or exclusion (absent link) between the different values. This representation resembles the information that we would recover if we build a network of variables from a covariance matrix: positive (negative) correlation arises when similar (dissimilar) values are found between two objects.

From the formal network in Fig 4 it is easy to observe that one of the values of the first variable, *d*_1_, is never observed, a fact that we can express with the proposition:

$$\begin{array}{c} \text{Ext}(d_{1})=\emptyset . \end{array}$$

What this proposition simply states is that, in order to identify that a given microstate belongs to this system, it is necessary to evaluate that the value measured at the first component of the system is different from zero. In other words, as soon as we reject the hypothesis *H*_0_: "*d*_1_ is observed", we will be confident of the fact that we are dealing with a microstate contained in *S*1. Similarly, to propose a generative model of the states, we just need to fix the first variable to one for every state and randomly generate values for the other two variables.

#### System with two constraints of scope two (S2)

We now select four microstates that are the result of imposing two constraints over two pairs of variables, which leads to the set {*μ*_1_, *μ*_4_, *μ*_5_, *μ*_8_} (see Table 3).

**Table 3:** 3-bit microstates of a system with two constraints of scope two.

{*μ*_E_}
*μ*_1_ = (1, 1, 1)
*μ*_4_ = (0, 0, 1)
*μ*_5_ = (0, 1, 1)
*μ*_8_ = (0, 0, 0)

We look for a compact description following the same reasoning as for S1. The expanded presentation of the system reads

$$\begin{array}{c} \Omega =\mu_{1}\vee \mu_{4}\vee \mu_{5}\vee \mu_{8}=\left(c_{1}\wedge c_{2}\wedge c_{3}\right)\vee \left(d_{1}\wedge d_{2}\wedge c_{3}\right)\vee \left(d_{1}\wedge c_{2}\wedge c_{3}\right)\vee \left(d_{1}\wedge d_{2}\wedge d_{3}\right). \end{array}$$

We start by looking for a simplified expression for *μ*_4_ ∨ *μ*_5_:

$$\begin{array}{ccc} \mu_{4}\vee \mu_{5}=\left(d_{1}\wedge d_{2}\wedge c_{3}\right)\vee \left(d_{1}\wedge c_{2}\wedge c_{3}\right) & = & d_{1}\wedge \left(d_{1}\vee c_{2}\right)\wedge \left(d_{1}\vee c_{3}\right)\\ & & \wedge \left(d_{2}\vee d_{1}\right)\wedge \left(d_{2}\vee c_{2}\right)\wedge \left(d_{2}\vee c_{3}\right)\\ & & \wedge \left(c_{3}\vee d_{1}\right)\wedge \left(c_{3}\vee c_{2}\right)\wedge c_{3}\\ & = & d_{1}\wedge c_{3} \end{array}$$

and the simplification is made after *d*_1_ and *c*_3_ absorbed several terms, and again neglecting those that are trivially true by construction. Next, we would like to compress states *e*_1_ ∨ *e*_8_, but this is not possible: by expanding the expressions it is possible to rearrange them, but there is no reduction in the end (not shown). The reason is that, if only these states would be observed, a global constraint should be acting on all three variables simultaneously, thus limiting their behaviour, and it is not possible to express with this formalism such a constraint except by presenting the states themselves. We will further explore this question in our last example below. For the present example, the description of the S2 system would be:

$$\begin{array}{ccc} \Omega & = & \mu_{1}\vee \mu_{4}\vee \mu_{5}\vee \mu_{8}=\left(c_{1}\wedge c_{2}\wedge c_{3}\right)\vee \left(d_{1}\wedge c_{3}\right)\vee \left(d_{1}\wedge d_{2}\wedge d_{3}\right) \end{array}$$

The expression achieved is an unambiguous compact description of S2, and it would be interpreted in a model as follows (reading first the left parenthesis): either the subject observes all three states ON (1,1,1), OR (s)he observes OFF in the first position and OFF in the third one (0,?,1) OR all three are OFF (0,0,0). The same result would be obtained reading the expression in any other order: independently of the path followed, a decision tree representing the constraints of the system can be established. To recover all four states we can develop a generative model applying the rules proposed and drawing random values in the positions that, when the leaves of the decision tree are reached, remain undetermined (as it was for the second position in (0,?,1)). Note that we have lost again information in the compact representation achieved, although not as dramatically as in the previous example, because there are more constraints and they have a larger scope.

**Fig 5:**
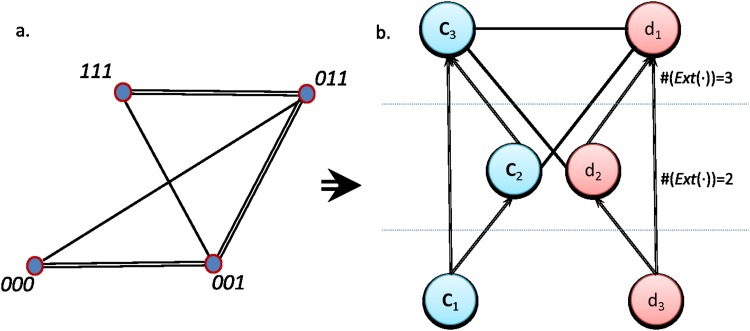
Representations of a three bits system with two constraints of scope two. (Left) In the concrete network, each node represents a microstate and it is linked with another microstate if they share the same observation for any component, where the number of links represent the number of concepts shared. (Right) Network of concepts extracted from the analysis of the microstates. Two links *c*_*i*_ and *c*_*j*_ are linked with a directed edge if Ext(*c*_*i*_) ⊆ *Ext*(*c*_*j*_) and with an undirected link if Ext(*c_i_*) ∩ Ext(*c_j_*) ≠ ∅. The concepts are hierarchically ordered according to the cardinality of their extension, i.e. the number of microstates they map. In this example we identify the constraints observing those links that, despite of being viable, are absent. For instance, there is no link between *d*_3_ and *c*_2_.

Now, looking into the concrete representation and, again following the procedure of the previous example (see Fig 5), we observe that the disconnected components in the formal network lead us to identify the constraints, that can be expressed with the propositions:

$$\begin{array}{c} \begin{array}{cc} \text{Ext}(c_{1}\wedge d_{2})= & \emptyset \\ \text{Ext}(c_{\text{2}}\wedge d_{3})= & \emptyset \\ \text{Ext}(c_{1}\wedge d_{3})= & \emptyset \end{array} \end{array}$$

It is easy to observe that one of these constraints is redundant. Given that *c*_2_ and *d*_2_ cannot be observed simultaneously, if *c*_1_ is observed it means that *c*_2_ is also observed and thus *d*_3_ cannot be observed. And the other way around, if *d*_3_ is observed *c*_2_ will not be observed and thus *c*_1_ cannot be observed. Therefore, the third constraint Ext(*c*_1_ ∧ *d*_3_) = ∅, is a consequence of the other two, and we can just speak about the constraints of the system with two propositions.

#### System with a single constraint of scope three (S3; the parity bit system)

Our last example is a set of microstates having an even number of *ON* bits, i.e. a single constraint involving all three components. This system has been previously introduced by Bar-Yam as a toy example of the particular type of emergent behaviour we introduced above called *strong emergence* [14]. For this system, given that we find two random values in two randomly selected bits, the third bit is constrained in such a way that the number of bits in the microstate is always odd. This rule is used in the control of message transmission, where the last bit (called the parity bit) is used to monitor the presence of errors in the chain transmitted. Note that we are not interested in understanding the system under this engineering perspective, as it provides already a rather *ad hoc* explanation on how the system is built [31] and, in this work, we assume no *a priori* knowledge of the underlying mechanisms generating the observation. It is just one possible observation that will be analysed as in the previous examples. The microstates we will consider are {*μ*_1_, *μ*_2_, *μ*_3_, *μ*_4_}, as explicitly shown in Table 4.

**Table 4:** 3-bit microstates of a system with a single constraint of scope three.

{*μ*_E_}
*μ*_1_ = (1, 1, 1)
*μ*_2_ = (1, 0, 0)
*μ*_3_ = (0, 1, 0)
*μ*_4_ = (0, 0, 1)

Again, we follow the same reasoning as in previous examples

$$\begin{array}{c} \Omega =e_{1}\vee e_{2}\vee e_{3}\vee e_{4}=\left(c_{1}\wedge c_{2}\wedge c_{3}\right)\vee \left(c_{1}\wedge d_{2}\wedge d_{3}\right)\vee \left(d_{1}\wedge c_{2}\wedge d_{3}\right)\vee \left(d_{1}\wedge d_{2}\wedge c_{3}\right) \end{array}$$

We look for a compact description of *e*_1_ ∨ *e*_2_:

$$\begin{array}{ccc} e_{1}\vee e_{2}=\left(c_{1}\wedge c_{2}\wedge c_{3}\right)\vee \left(c_{1}\wedge d_{2}\wedge d_{3}\right) & = & c_{1}\wedge \left(c_{2}\vee d_{3}\right)\wedge \left(c_{3}\vee d_{2}\right). \end{array}$$

Proceeding similarly with *e*_3_ and *e*_4_ yields

$$\begin{array}{ccc} e_{3}\vee e_{4}=\left(d_{1}\wedge c_{2}\wedge d_{3}\right)\vee \left(d_{1}\wedge d_{2}\wedge c_{3}\right) & = & d_{1}\wedge \left(c_{2}\vee c_{3}\right)\wedge \left(d_{2}\vee d_{3}\right), \end{array}$$

and the description of the whole system would be

$$\begin{array}{ccc} \Omega & = & e_{1}\vee e_{2}\vee e_{3}\vee e_{4}=[c_{1}\wedge \left(c_{2}\vee d_{3}\right)\wedge \left(c_{3}\vee d_{2}\right)]\vee [d_{1}\wedge \left(c_{2}\vee c_{3}\right)\wedge \left(d_{2}\vee d_{3}\right)]. \end{array}$$

The expression provides a kind of “bottom-up” description of the constraints of the system, that would allow us again to build a decision tree to propose a model. For instance, reading the expression from left to right we would say that if we observe ON in the first position (1,?,?) then, either we observe (1,1,?) and then (1,1,1), or (1,0,?) and then (1,0,0). The two remaining states would be obtained if OFF is observed in the first position. However, the expression achieved is not a compact description. The reason is that, even if in the search for a reduced description we absorbed a large number of terms, these were redundant, because if we expand the expression found we exactly recover the definition of the four microstates. For instance, expanding the expression between the first square brackets we get the first two states:

$$\begin{array}{ccc} c_{1}\wedge \left(c_{2}\vee d_{3}\right)\wedge \left(c_{3}\vee d_{2}\right) & = & c_{1}\wedge (\left(c_{2}\wedge c_{3}\right)\vee \left(d_{2}\wedge c_{3}\right)\\ & = & \left(c_{1}\wedge c_{2}\wedge c_{3}\right)\vee \left(c_{1}\wedge d_{2}\wedge d_{3}\right)=e_{1}\vee e_{2}, \end{array}$$

and the other two are recovered from the second pair of brackets. Since a global constraint is acting on the system, we obtain that all the possible values of the variables are entangled. Therefore, the description of the constraint cannot be compressed, and hence it requires as much information as the presentation of the system itself, we are somehow “walking in circles” when we look for a description of the global constraint. We may say that we reached a more “expressive” description of the system, because we observe the relation between the values of the properties rather than the properties themselves but, since it is not compact, it may be also seen as an impredicative definition of the system’s constraints: we are defining the constraints with an expression that, rearranged, leads to the presentation of the system itself. This fact strongly ressembles Rosen’s closure of efficient causation but, while the relational closure in his approximation focuses on the causal relationships between the concepts, we still have no clue about the nature of the constraints. However, it seems remarkable that global constraints seems to induce a closure from the point of view of the relation between the concepts what, in the jargon used by Rosen, would mean that the system is complex, as opposed to a mere complicated system.

**Fig 6:**
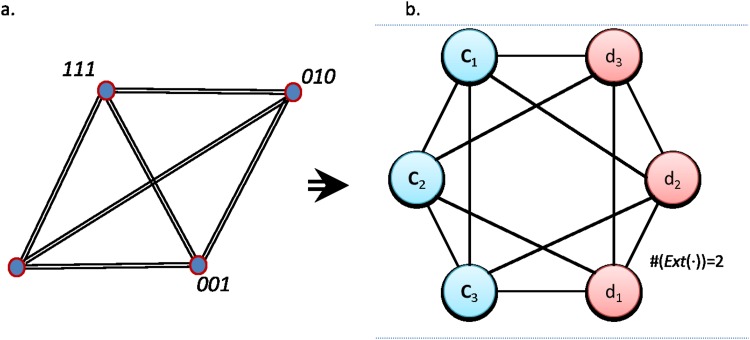
Representations of a three bits system with one constraint of scope three. (Left) In the concrete network, each node represents a microstate and is linked with another microstate if they share the same observation for any component, where the number of links represent the number of concepts shared. (Right) Formal network of concepts extracted from the analysis of the microstates. Two links *c*_*i*_ and *c*_*j*_ are linked with a directed edge if Ext(*c*_*i*_) ⊆ *Ext*(*c*_*j*_) and with an undirected link if Ext(*c_i_*) ∩ Ext(*c_j_*) ≠ ∅. The graph of concepts is equivalent to the graph we would obtain for a free system, being just observed a reduction in the number of objects mapped by each concept (from #(Ext(⋅)) = 4 towards #(Ext(⋅)) = 2). It reflects the notion that the system has “no borders”.

This is readily apparent when we look at the network representations (see Fig 6), because the formal network intuitively resembles a sphere in the sense that there are no “borders” –i.e. disconnected concepts from which propositions about the constraints can be derived by simple inspection, nor concepts mapping the empty set–. Thus, the identification of constraints is more difficult than in the previous examples also under these representations. Indeed, it seems possible to identify that there is a global constraint only because we already know the viable values. Considering all eight microstates of the free-of-constraints system highlights a lower cooccurrence of the different values of the three variables, but there will be no differences in the final network topology we obtain. This fact would be also observed in the marginal probabilities, as no bias will be observed for the free system nor for the parity bit system.

The comparison with the free system lead us to conclude again that in order to speak about the system we need to write down all the microstates that are not observed:

$$\begin{array}{c} \begin{array}{cc} \text{Ext}(d_{1}\wedge c_{2}\wedge c_{3})= & \emptyset \\ \text{Ext}(c_{1}\wedge d_{2}\wedge c_{3})= & \emptyset \\ \text{Ext}(c_{\text{1}}\wedge c_{2}\wedge d_{3})= & \emptyset \\ \text{Ext}(d_{\text{1}}\wedge d_{2}\wedge d_{3})= & \emptyset \end{array} \end{array}$$

which requires the same amount of information as presenting the system itself, as was pointed out above.

We conjecture that, for systems with global constraints, it is not possible to derive a compact description within this formalism. Of course, we may think that our formalism is simply insufficient and that there may be other more sophisticated formalisms that would be able to express the constraints in S3 under a compact description. For instance, there may be a function *f* which, given a description of a microstate *e*_*i*_, returns a new *type* of concepts $\tilde{c}=f\left(e_{i}\right)$ that is able to differentiate the microstates of S3 from the rest. Let’s see this with an example. We can characterize the three above examples with their probability distributions:

$$\begin{array}{cc} P\left(\mu \right)=\delta \left(x_{1},1\right)/2^{n+1} & \left(S1\right)\\ P\left(\mu \right)=\delta \left(\delta \left(H_{12}+1,1\right),H_{23}+1\right)/2^{n+1} & \left(S2\right)\\ P\left(\mu \right)=\delta \left({\text{mod}}_{2}\left(\sum_{i}x_{i}\right),1\right)/2^{n+1} & \left(S3\right) \end{array}$$

where *x*_*i*_ is the value of the bit *i*, *n* is the number of bits, *δ*(a, b) is the Kronecker’s delta, *H*_*ij*_ = *H*(*x*_*i*_ − *x*_*j*_ − 1) is the Heaviside function and mod_2_(⋅) is the module two function. In principle, there is no reason to think that the probability distribution of S3 is more complex than those of S1 and S2. To say this there should be any objective reason showing, for instance, that the use of a summation operator and a module function is more complex than applying two Heaviside functions.

To circumvent these issues, measures like the algorithmic information complexity, also known as Kolmogorov complexity, have been proposed [32]. This is the spirit in which we developed the present proposal. We believe that the fact that the formalism we are using here has well defined limits (arising from the rather limited repertoire of operations allowed), is not a drawback but an advantage to fairly compare different systems. Furthermore, when we reach a limit, as it is the case for S3, we have a clear intuition of which are the ontological properties behind our epistemological limitation, in this case the existence of a global constraint. For instance, a desirable property of a framework should be that, if we increase the system’s size, our ability to describe the constraints of a larger system should change according with their number and scope. If we imagine how our examples will increase the number of microstates when the number of components *N* increases, both S1 and S3 will increase as 2^*N*−1^ while S2 increases as *N* + 1. According to our formalism, the length of the description of the system will change with increasing *N*. For instance, the number of propositions mapping the formal and concrete state of the type Ext(*c*) = ∅ we need in order to describe the constraints in the system remains equal to one for S1, increases as *N* − 1 for S2, and as 2^*N*−1^ for S3. We believe that this finding is remarkable and resembles the problems identified by Tsallis [33] with the extensivity of the entropy. In the following sections, we exploit it to provide a quantification of emergence compatible with the scientific method.

### Quantification of emergence

According to the above results, the number and scope of the constraints are ontological properties that determine our epistemological ability to achieve a compact description, which we raised as a necessary condition to delineate a model aimed at explaining an emergent macroscopic property. If, as is the case with the formalism we selected, these constraints are fairly comparable among systems, then the amount of information that codifies its description seems to be a natural way to quantify emergence. It is, however, difficult to imagine that there exist a framework general enough to perform such quantification in real systems.

In this section we propose a series of concepts that we believe are compatible with computational modelling of complex systems, and that are likely independent of the system used and modelling approach followed. We start by supposing that we are dealing with a model of an emergent behaviour, and that our strategy consists of performing *interventions* to the system such that variables in the model are substituted for variables free of constraints. We then monitor what is the relative change in our ability to predict the system’s behaviour after the intervention.

#### Coverage excess

Intervention is a basic strategy to link computational modelling with the scientific method [34]. When we neglect any variable describing a system, we reduce our predictive power if the variable is constrained with others, as the latter will lose specificity and so there will be more states of the system compatible with the remaining constraints. We can quantify the uncertainty we generate when we lose constraints; meaning that we can relate the causal effects a component has on the other components according to the notion of Granger causality [35].

**Fig 7:**
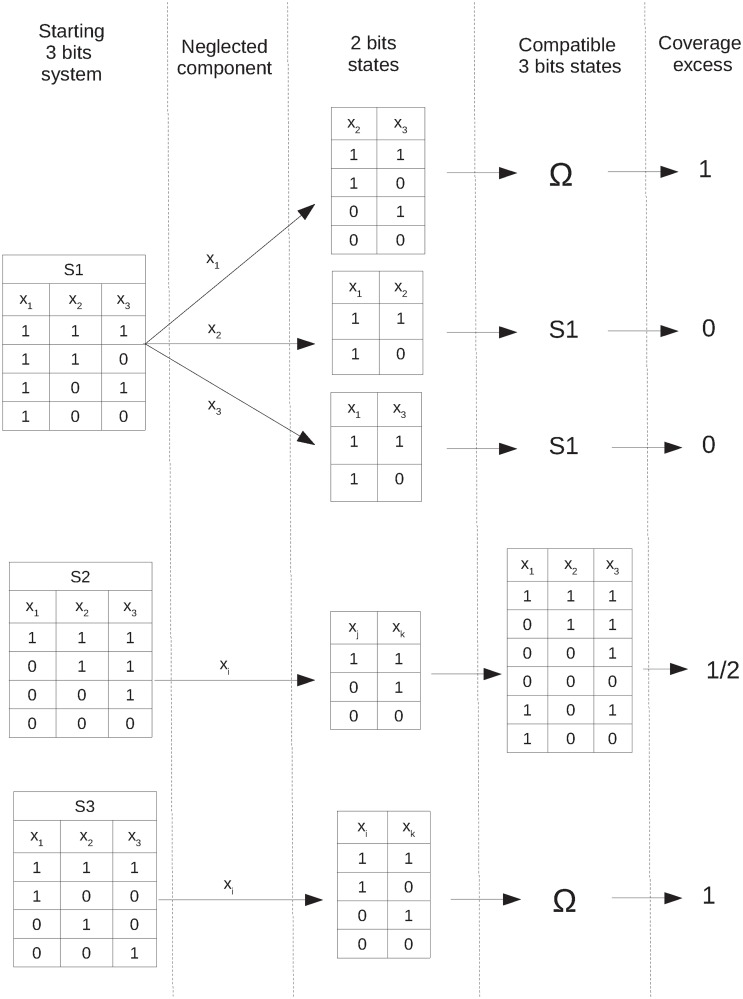
Scheme illustrating the definition of coverage excess. The scheme is divided in five columns (1-5) that we describe from left to right. (1) The 3-bit systems under analysis in the main text are shown. If we intervene in the systems neglecting one component (2) we will obtain a set of 2-bit states (3). For the system S1, removing *x*_1_ lead to different states than if *x*_2_ or *x*_3_ are removed, while S2 and S3 lead to the same states independently of the component removed (see Main Text for details). From the 2-bit states, we recover the neglected component keeping it free of any constraint, which leads to a number of compatible 3-bit states (4). In the last column we show the result for the coverage excess obtained from this procedure using Eq 8. The final value for the coverage excess of the system will be the average among the values obtained from the different interventions.

We illustrate the proposed method in Fig 7 by considering the 3-bit synthetic examples. Starting from a 3-bit system, we neglect one of the variables, and then we explore which are the 2-bit states recovered. Returning to the system with the chosen variable neglected, but allowing it to now obtain any viable value (i.e. free of constraints), we will be able to build from these 2-bit states a number of 3-bit states. By doing this systematically for all the variables, we can infer what is the influence of the underlying constraints. For instance, for S1, if we neglect the first component *x*_1_, which is the source of the constraint, we obtain all possible states of a 2-bit system containing components *x*_2_ and *x*_3_. This is because there is a single constraint in the system and it was deleted, hence we can recover the whole phase space Ω when we build up all the 3-bit states compatible with these 2-bit states. On the other hand, if we neglect any of the other two components, *x*_2_ or *x*_3_, the constraint still remains in the first component *x*_1_. Therefore, if we look for the 3-bit states compatible with these 2-bit states, we will be constrained to come back to the original system S1. For the system S2, irrespective of the component neglected we will reach the same 2-bit states. From them, we obtain not only the original 3-bit states but two more, which means that we have lost one constraint every time, but the other one is still active. For S3, irrespective of the component neglected, we obtain all possible two bits states, and hence the whole phase space will always be recovered, meaning that the constraint is always completely lost, thus pointing towards the existence of a global constraint.

With these kinds of interventions we can quantify how the original system is *covered in excess* when an intervention takes place. Formally, let’s call *U* the subset of concepts contained in the focus *F* describing a set of microstates *S* = {*μ*} containing *N* components in the phase space Ω, which we know is associated with a target macroscopic property. For simplicity, suppose that each component *i* is fully characterized by a single variable *x*_*i*_. Next, remove any of the *x*_*i*_ variables and consider a new set of microstates *S*′ of a system with *N* − 1 components. Let’s then denote as *V*(*x*_*i*_) the subset of concepts describing the set of microstates *S*′′ of a system with *N* components obtained by adding a new unconstrained component $x_{i}^{\prime }$ to *S*′. We say that the coverage excess Ξ of *S* induced after neglecting the variable *x*_*i*_ and introducing back the component $x_{i}^{\prime }$ is the quantity

$$\begin{array}{c} \Xi \left(x_{i}\right)=\frac{\#\left(\text{Ext}\left(V\left(x_{i}\right)\right)\right)-\#\left(\text{Ext}\left(U\right)\right)}{\#(\text{Ext}(F))-\#(\text{Ext}(U))}=\frac{\#\left(S^{\prime \prime }\right)-\#\left(S\right)}{\#(\Omega )-\#(S)} \end{array}$$ (8)

where the function #(⋅) returns the number of microstates contained in the set. This quantity takes a value of zero when the states recovered are the same as the original ones, and it is equal to one when the whole phase space is recovered. To consider a single value for the coverage excess we will average the result of the intervention over all variables:

$$\begin{array}{c} \langle \Xi \rangle =\frac{1}{N}\sum_{i}\Xi \left(x_{i}\right) \end{array}$$ (9)

If the system is very large an exhaustive computation may be unfeasible, and a random sampling of the different components or more complex interventions such as the removal of several variables would be needed. We indicate this average over interventions with the brackets 〈⋅〉. The examples explored for the three bits system results in 〈Ξ〉_*S*1_ = 1/3, 〈Ξ〉_*S*2_ = 1/2 and 〈Ξ〉_*S*3_ = 1. The coverage excess reflects the vulnerability of the system to this intervention and, as the effects link the interventions with the number and scope of the constraints present in the system, it provides a mechanism to differentiate between upward and downward causation [36]. The system S1 is very vulnerable if *x*_1_ is neglected, but it is not affected at all if any other variable is neglected, and thus there is upward causation from the first variable to the whole system. On the other hand, the system S3 is very vulnerable as the coverage excess is maximum irrespective of the variable over which we intervene, thus highlighting that there is a global constraint affecting the system downwards. We also note that these values will scale differently with the system’s size. While for *S*1 〈Ξ〉_*S*1_ → 0 when *N* → ∞, for *S*3 it will remain constant and equal to one. Finally, we consider the particular case in which we are already dealing with a subset of microscopic concepts *U* such that an emergent process described by the macroscopic concept $\hat{c}$ is perfectly covered, i.e. such that $\text{Ext}\left(U\right)=\text{Ext}\left(\hat{c}\right)$. In this case, we will say that the Eq 9 provides *the coverage excess of the emergent property* $\hat{c}$, that we denote as $\langle \hat{\Xi }\rangle$.

#### Loss of traceability and emergence strength

The sensitivity of the model when an intervention over the system takes place suggests that systems with higher coverage excess will be more difficult to analyse. This difficulty can be combined with the notion of traceability we proposed above. If we have a perfectly traceable system, we can quantify how much traceability we lose after intervention with the *loss of traceability*

$$\begin{array}{c} ϒ=1-\langle \hat{\Xi }\rangle . \end{array}$$ (10)

With this quantity, we can express those systems that are easily covered in excess have low traceability and the other way around. Therefore, the associated macroscopic property will be difficult to explain, from which the following definition for the *emergence strength* *σ* of the emergent property arises naturally

$$\begin{array}{c} \sigma =-\text{log}(ϒ). \end{array}$$ (11)

We haven taken the logarithm of the traceability to represent the emergence strength as a dissimilarity between a state of perfect knowledge of the system (complete traceability) and the state after intervention. If, after intervention, we still remain in a situation of perfect traceability, this dissimilarity will be zero. On the other hand, if we completely lose all the information about the system in a way that we recover an unconstrained phase space, this dissimilarity will be infinite, thus reflecting some epistemological gap for these systems.

With these definitions we expect to reconcile different positions on whether the origin of emergence is epistemological or ontological: even if we deal with a perfectly traceable system, which is therefore epistemologically accessible, we can still see that there are systems that are more inaccessible than others, and there are ontological reasons for that: the type of constraints involved in the system. For these systems, until perfect traceability is attained, we will probably be tempted to say that they are epistemologically inaccessible, and that there is a strictly ontological and not epistemological reason for that. But it is a combination of both: there is an ontological reason why their emergence strength is so high that hinders an epistemologically accessible (microscopic) compact description, but this doesn’t mean that such a description cannot be achieved at some point. Of course, we should keep in mind that achieving a compact description does not mean that we have a full mechanistic understanding of the process: we still need to develop a model in the sense introduced here, that would interpret the constraints and generate the microcopic phase space. If the constraints are very complex the implementation of constraints may never be feasible. Furthermore, even if a satisfactory generative model is developed, it does not mean that we are able to generate the macroscopic emergent pattern with a model. This would require a final process to decode the formal model, *sensu* Rosen [28], which may further require a complex experimental setup (the decoding step shown in Fig 1).

Given that during the process of researching an emergent property there are different steps we could use to evaluate the accessibility of the underlying process, we propose to focus upon the analysis of the patterns obtained from the experimental data as the starting step. This may help to reconcile the definition of weak and strong emergent properties [14] using the emergent strength: if the emergence strength is infinite we deal with a strongly emergent pattern, whereas if the value is finite we deal with a weak emergent pattern with the associated strength as an indicator of how difficult it is to achieve traceability. We conjecture that further difficulties in computational or experimental modelling will likely go hand in hand with difficulties in the determination of the pattern’s traceability.

Of course, we cannot discount the possibility that there exist systems which are epistemologically inaccessible. This may be the case for quantum or computational systems—for which some of the definitions of weak and strong emergence where originally proposed—but not for many systems of scientific interest, where we believe that the situation is the one that we examined here: these systems are very large and they are under constraints large in scope. Note that it may be argued that, with the above definitions, the emergence strength cannot be determined unless we achieve perfect traceability. However, this is only true for strongly emergent properties because Eq 10 can be easily modified to consider an intermediate (incomplete) model, using only the expression in Eq 9 (and not $\langle \hat{\Xi }\rangle$), which will give us an estimate of the emergence strength. We expect that, for natural systems, there is a complex structure of constraints with different scopes, and we will be able to progressively discover this structure –possibly from low to high scopes– and so provide an estimate at any time.

## Discussion

In this article, we presented a novel approach to investigate the concept of emergence in complex systems. We tackled the problem through a constructive logical system that permits the investigation of the relationship between concepts and objects of observation [25]. In doing so, we focused upon a particular kind of system, which we believe are of much interest to the current discussion of emergence. We start by supposing that we are analysing a naturally occurring macroscopic emergent property, and not a purely computational one. In addition, we neglect any vitalism, which means simply accepting explanatory physicalism; in the words of Mitchell, what else could there be? [17].

We then assume that we are able to describe microstates of the system through experimental measurements. This implicitly assumes that we are able to differentiate the system from its background [21] and to provide a bottom-up characterization in terms of concepts associated with the elements that constitute the system [16], thus justifying the constructive approach. Nevertheless, we allowed for the possibility that we have no clue about the mechanistic processes underlying these observations, as often happens when a research program is in its infancy.

This fact differentiates this work from other theoretical approximations aiming to understand which features belong to systems exhibiting emergent properties, but that already assume that sufficent knowledge about the system exists so as to test its computational compressibility (Bedau [8]), to postulate the existence of a closure of efficient causation (Rosen, which requires determining the causal relationships [28]) or to intervene over a system for which we already have a mechanistic model (see for instance Hoel et al. [37]). However, this is typically not the case when in the early stages of research, and this is why believe our proposal may be helpful for a wide variety of scientists. Invoking a mild condition relating the macroscopic observation of an emergent property and the constrained walk of the system in a certain region of the phase space, we focus on systems from which we expect to find sufficient regularities in the analysis of their microstates so as to be able to build explanatory models, i.e. in potentially robust emergent systems, which we believe are of key interest to the scientific community [13].

We showed that building a microscopic model aimed to explain an emergent macroscopic observation requires identifying constraints in the viable values of the microscopic variables. Interestingly, we identified concept disjunction as the basic logic operation to find constraints. The search for similarity measures, dissimilarity measures or distances is an essential task in Biology and Ecology [38] aiming to understand, following a top-down approach, the information shared between the different observations. This probably explains the success of complex networks theory and its philosophical interest, or why methods comparing objects of observation, such as protein sequence alignments like BLAST [39], are among the most cited ever in the scientific literature [40]. In general, disjunction underlies dimensionality reduction techniques such as principal components analysis [41]. From the perspective of our framework, these are techniques aiming at obtaining a representation with the minimum number of concepts whose extension explains the full variability of the microstates. In this way, we are able to *talk* about the set of objects using a subset of concepts, which is essentially the task addressed by dimensionality reduction techniques, and that we defined here with the notion of compact description.

We applied these tools to three different ensembles of microstates of a 3-bit synthetic system. We observed that the scope of the constraints is the main difficulty in identifying them: the larger the scope of the constraint, the more difficult is to assess it. In particular, our method was unable to find a compact representation when the scope of the constraint has the same size as the system, which directly links the epistemological limitation of our framework with an ontological property of the system. We briefly considered other approximations, and were able to show that the number and type of constraints heavily influence the consequences that either an increase in system size or a loss of components may have upon our ability to identify them. This observation seems to be independent of the formalism used, and so will also be independent of any subjectivity induced by the formalism we chose here. Notably, we were able to express this observation in the concrete space; thus further research would be needed to find equivalent definitions in the formal space.

We also proposed a procedure based on the intervention of the observer on the system, thus compatible with the scientific method, to compute the loss of information experienced when we neglect components in the system. Given that the loss of information depends on the type of constraints present, we can quantify how difficult it is to achieve traceability between the microscopic and macroscopic description. The loss of traceability was then used as a quantity to establish a distance between perfect traceability and our knowledge of the system after systematic interventions, which was what we called the emergence strength.

We believe that, for the kind of systems we are interested in, the emergence strength paves the way for us to reconcile different notions of emergence. For natural systems, we aim to develop computational models to reproduce experimentally measured data and to then simulate the emergent process, and thus it is compatible with weak emergence. Nevertheless, we propose to combine the ability to build a computational model with the identification of constraints from experimental data, since the identification of constraints is where we start learning about the natural process we face. In this way, we focus on disentangling the number and scope of the constraints, whose complexity will determine its emergence strength.

We conjecture that, for systems with different types of constraints, those with smaller scope are identified first. Accordingly, if a system has only constraints with a large scope or there is a big gap with respect to those with smaller scope, it may be simply impossible at a certain state of knowledge to assess them, an example of which may be our current knowledge of consciousness. For these processes, the emergence strength may be so high that we would be justified in calling them strongly emergent processes. For such processes, it may be the case that not only is it impossible to decipher the constraints, but even if the microscopic constraints are deciphered, it may still not be possible to build an experimental setup for a model to recreate the observed emergent pattern, given the complexity of the environment in which the system should be embedded to reproduce such a constraint. Our definition seems to also be compatible with the classification proposed by de Haan [7], as the existence of a microscopic emergent conjugated causally affecting the macroscopic pattern (in the strongest version, consciously), can be understood in terms of a global constraint (as he suggests in the relationship between this type of emergence and downward causation). This is the case for living systems, where we believe strong emergence may be pervasive.

Our findings might be criticized as saying that describing emergence in terms of constraints provides a static description for systems that are intrinsically dynamic; an approach which may be thought of as a kind of ontological reductionism [17]. Note, however, that constraints may themselves be dynamic and, either their variation occurs on a longer timescale, or the constraints dynamics is itself sufficiently constrained, e.g. periodic conditions such as day-night or seasonal temperatures. This would also address potential criticisms regarding multiple realizability: similar microscopic patterns can be found for systems under similar constraints even if the particular realizations of the microstates are substantially different for each system. Multiple examples of this can be found in the literature. For example, the evolutionary process allow us to classify protein structures in clusters if they have global structural similarity (that result from similar physico-chemical constraints) even if they perform different functions [42]. Similarly, ecological patterns such as the nestedness found in ecological networks describe complex constraints in the way in which species interact, even if they are found in different ecosystems, from plant-pollinators [43] to host-virus systems [44].

If patterns observed in organisms are the consequence of natural selection, and global constraints are acting on individuals in the selection process, natural selection itself can be thought of as an expression of downward causation [45]. Interestingly, adaptation takes place when the organisms are able to predict and overcome environmental changes, and predictability is a consequence of the amount of structured information that exists in the environment [46]. As a corollary, the ability of organisms to modify the environmental constraints to make them more predictable, enhances the organisms’ potential for adaptation. But, organisms share their environment with other organisms, and thus ecological interactions are fundamental to the adaptive process. This picture, in its stronger version, in which the influence of ecological interactions is so important that the notion of an individual as object of selection is challenged, becomes increasingly important in current research, particularly in the microbial world (see for instance [47]).

To illustrate this point, consider the following example proposed in [48], in which we consider one individual for which its fitness *f*_*i*_ can be decomposed into two components, where the first component reflects the fitness $f_{ij}^{\text{int}}$ of the individual as a consequence of its ecological interactions with other species *j*, and the second its fitness $f_{i}^{\bar{\text{int}}}$ due to any other process, i.e. $f_{i}=f_{ij}^{\text{int}}+f_{i}^{\bar{\text{int}}}$. Now consider a particular example, in which two individuals belong to two different species, *a* and *b*, interacting mutualistically. The effect of the interaction on the fitness *f*_*i*_ would be positive through an increase reflected in the term $f_{ij}^{\text{int}}$. Finally, think of an evolutionary event which becomes fixed in the population of species *a* affecting its fitness, $f_{a}\to {\hat{f}}_{a}$, in such a way that the new fitness ${\hat{f}}_{a}<f_{a}$ and, in particular, ${\hat{f}}_{a}^{\bar{\text{int}}}=f_{a}^{\bar{\text{int}}}$ but ${\hat{f}}_{ab}^{\text{int}}<f_{ab}^{\text{int}}$. This means that the fitness of species *b* due to the interaction with species *a* will also be affected after the evolutionary event and thus there will be a change in the selection pressure on the regions of the genomes of both species codifying the traits needed for the interaction. Furthermore, if we consider an extreme scenario in which $f_{ab}^{\text{int}}\gg f_{a}^{\bar{\text{int}}}$ and $f_{ba}^{\text{int}}\gg f_{b}^{\bar{\text{int}}}$ –that may be the case for auxotrophs (see a synthetic ecological experiment in [49])– the relevance of these coevolving regions in the evolutionary process would be so important that the concept of an object of selection should be revisited [50]. In particular, it might be more appropriate to frame the evolution of both species by considering them as some sort of multicellular species. In this sense, even if the individual is still the main object of selection, it becomes entangled with an object of selection determined on a larger scale, which is the consortia of species. Furthermore, if this consortia acquires new functionality that make its members selectively advantageous in line with Kim [5], we may say that a new object of selection emerges. If that were the case, the single species’ individuals would be constrained downwards by their fitness dependence with respect to the consortia they belong to, and the consortia is itself influenced upwards by the individual species. In the same way that we admit the existence of different levels of organization of living beings that depend on a hierarchy of constraints, we should also consider the possibility that individuals belong to different objects of selection influencing their fitness to different degrees. In this way, the term upwards (downwards) causation would be used in this context as the effect of constraints acting on a given level has ramifications for objects of selection on upper (lower) levels.

This perspective probably reflects the interest of modern science on mutualistic interactions and its relation with emergent processes [4], although mutualism may not be necessary to derive a measure of community-level fitness [51]. The determination of fitness above the individual level may be seen as a form of self-determination that would engage with the concept of closure of efficient causation. And in the same way that it is possible to argue that complete or perfect closure exists for any living being, it is also possible to argue for the concept of the object of selection to be a closed concept and reality. This is probably why it is aimed at extending the concept of closure to an ecological context [52]. A rigid definition of closure or of an object of selection might be an acceptable concept in an ideal thermodynamical scenario of natural selection [53] –and thus theoretically interesting– but it may perhaps be a good idealization of the limits of biological organization, like simple cells or the whole biosphere. But these concepts become blurred in complex ecosystems in natural environments. We believe that there is no need to invoke any teleological principle [54]. Only in an ideal scenario in which life evolves spontaneously following thermodynamical principles may one envisage a teleological ideal in which biological organization maximizes any entropic or energetic principle [55], or arbitrary fitness. It can be argued that this scenario is true in general. A simple counterexample may be found in the way in which human beings not only do not optimize any spontaneous physico-chemical principle from which life may have emerged, but rather generate a process that may eventually lead to extinction.

In summary, we believe that the formalism introduced here improves our ability to synthetically understand complex systems. We believe that it could also be used to tackle other challenging questions, and thus we hope that our effort will stimulate both scientific and philosophical discussion. Looking for fresh formal approaches to talk about philosophical questions is particularly important because formally shaping our philosophical knowledge is a way to create new bridges between science and philosophy. This would be probably good news for science, as the benefits of philosophy seem to be, for current scientists, left behind.
